# Metagenomic Insights into the Functional Profiles of Carbon, Nitrogen, and Phosphorus Cycles in Yuncheng Salt Lake Under Different Salinity Gradients

**DOI:** 10.3390/microorganisms14091937

**Published:** 2026-09-02

**Authors:** Jing Yang, Zhuo Wang, Chuanxu Wang, Yunjie Li, Yajie Niu, Jia Feng, Shulian Xie, Xin Li

**Affiliations:** 1 Shanxi Key Laboratory of Yuncheng Salt Lake Ecological Protection and Resource Utilization, Life Sciences Department, Yuncheng University, Yuncheng 044000, China; wangzhuojs@ycu.edu.cn (Z.W.); wangchuanxu@ycu.edu.cn (C.W.); liyunjie@ycu.edu.cn (Y.L.); niuyajie@ycu.edu.cn (Y.N.); 2 School of Life Science, Shanxi University, Taiyuan 030006, China; fengj@sxu.edu.cn (J.F.); xiesl@sxu.edu.cn (S.X.); 3 Department of Biology, Xinzhou Normal University, Xinzhou 034000, China

**Keywords:** salinity, microorganisms, metagenomics, element cycles, salt lake

## Abstract

Salinity is a key driver of microbial community structure and function in salt lake ecosystems, yet how it shapes functional genes involved in carbon (C), nitrogen (N), and phosphorus (P) cycling remains poorly understood. We collected metagenomic samples along a natural salinity gradient in Yuncheng Salt Lake and examined how salinity was associated with microbial taxonomic and functional diversity and with C, N, and P cycling genes. Both diversity metrics decreased significantly with increasing salinity and were positively correlated with each other. The composition and abundance of C, N, and P cycling genes differed significantly among the low-, medium-, and high-salinity groups. In carbon cycling, most carbon fixation genes were more abundant at higher salinity, whereas most carbon degradation genes were less abundant; within carbon fixation, reductive tricarboxylic acid (rTCA) cycle and Calvin cycle gene abundances were higher. In nitrogen cycling, nitrogen mineralization and assimilation genes were significantly more abundant. In phosphorus cycling, transporter and pyrimidine metabolism genes were more abundant, whereas the relative contribution of purine metabolism genes declined. Co-occurrence network analysis revealed dense positive co-occurrence associations among C, N, and P cycling genes, with *mer*, *GLU*, and *ppk1* as highly connected genes. Mantel tests identified salinity and pH as the primary environmental factors associated with functional gene variation. These results suggest that salinity may regulate C, N, and P cycling genes partly by reshaping microbial community structure in salt lake ecosystems.

## 1. Introduction

Inland salt lakes account for approximately 44% of the world’s inland water area. Owing to their unique hydrogeochemical characteristics, including high salinity, intense evaporation, and frequent wet-dry cycles, they constitute a dynamic yet often overlooked interface in global element cycles [1]. Salinity is the most critical environmental filtering factor in salt lake ecosystems, profoundly regulating the structure and functional traits of microbial communities by inducing osmotic stress, altering nutrient availability, and influencing microbial metabolic activity. Increased salinity can alter the ionic balance of H^+^ and OH^−^ on cell membranes, thereby affecting microbial growth and metabolism [2]. In high-salinity environments, microorganisms regulate cellular osmotic pressure by accumulating compatible solutes, such as amino acids, sugars, and polyols, to maintain normal physiological functions [3]. Increased osmotic pressure kills salinity-sensitive species, whereas halophiles proliferate, thereby reducing bacterial diversity [4].

Salinization affects not only microbial community composition [5,6] but also, through consequent changes in microbial activity, the functional genes involved in carbon degradation and nitrogen cycling [7] and in nutrient cycling more broadly [8,9]. Previous studies reported that increased salinity significantly suppressed the abundance and expression of genes related to carbohydrate and methane metabolism [10]. In contrast, a study on the Qinghai–Tibet Plateau reported that salinity promoted functional genes associated with carbon degradation, denitrification, and sulfur oxidation [11]. These conflicting results may be attributed to differences in climatic conditions, ecosystems, or salinity ranges among studies. Physicochemical factors other than salinity also influence the distribution of microbial community functions, and the primary drivers under different ecosystem conditions remain controversial. For example, in saline ecosystems of Northeast China, bacterial community composition and diversity were significantly influenced by pH, electrical conductivity, Na^+^, K^+^, Cl^−^, and CO_3_^2−^ [8], whereas oxygen and pH were the key drivers of microbial community [12]. As important inland water bodies in arid and semi-arid regions, salt lakes are unique extreme environments. Their high-salinity, oligotrophic habitats shape distinctive biological community structures and drive element cycling patterns that differ markedly from those of freshwater ecosystems. Which factors most strongly shape microbial community structure and function in these ecosystems therefore remains unresolved.

Carbon, nitrogen, and phosphorus are the core biogenic elements supporting microbial growth and metabolism, and their biogeochemical cycles are highly coupled under microbial control. Organic carbon mineralization provides electron donors for denitrification; nitrogen availability regulates microbial phosphorus uptake and phosphatase secretion; and phosphorus, as a key component of nucleic acids, ATP, and phospholipids, constrains biomass synthesis and energy metabolism [13,14,15]. Under high-salinity stress, microorganisms synthesize large amounts of compatible solutes to maintain cellular osmotic balance, a process that consumes substantial carbon skeletons and reduced nitrogen and may redistribute C, N, and P metabolic fluxes. Nevertheless, research on these elemental cycles in high-salinity environments remains limited, and the distribution patterns and coupling relationships of C, N, and P metabolic functional genes along salinity gradients have not been systematically characterized in salt lake ecosystems.

Yuncheng Salt Lake is located in the arid-semi-arid region of southern Shanxi Province (34°36′–35°00′ N, 110°50′–111°15′ E) and is one of the earliest salt lakes developed in China’s history. As a typical sodium sulfate-type salt lake with marked spatial heterogeneity in salinity, it is influenced by both natural evaporation and human salt harvesting, making it an ideal natural laboratory for examining how salinity and other environmental factors shape microbial community structure and function. Here, we combined metagenomic sequencing with physicochemical analysis to (i) characterize microbial community succession along the salinity gradient; (ii) determine how key functional genes involved in C, N, and P cycling respond to salinity; (iii) identify the environmental factors driving the distribution of microbial community structure and functional genes; and (iv) reveal the coupling relationships among C, N, and P cycling genes. Our results deepen the understanding of how microorganisms drive element cycling in extremely high-salinity environments.

## 2. Materials and Methods

### 2.1. Study Area and Sample Collection

**Figure 1:**
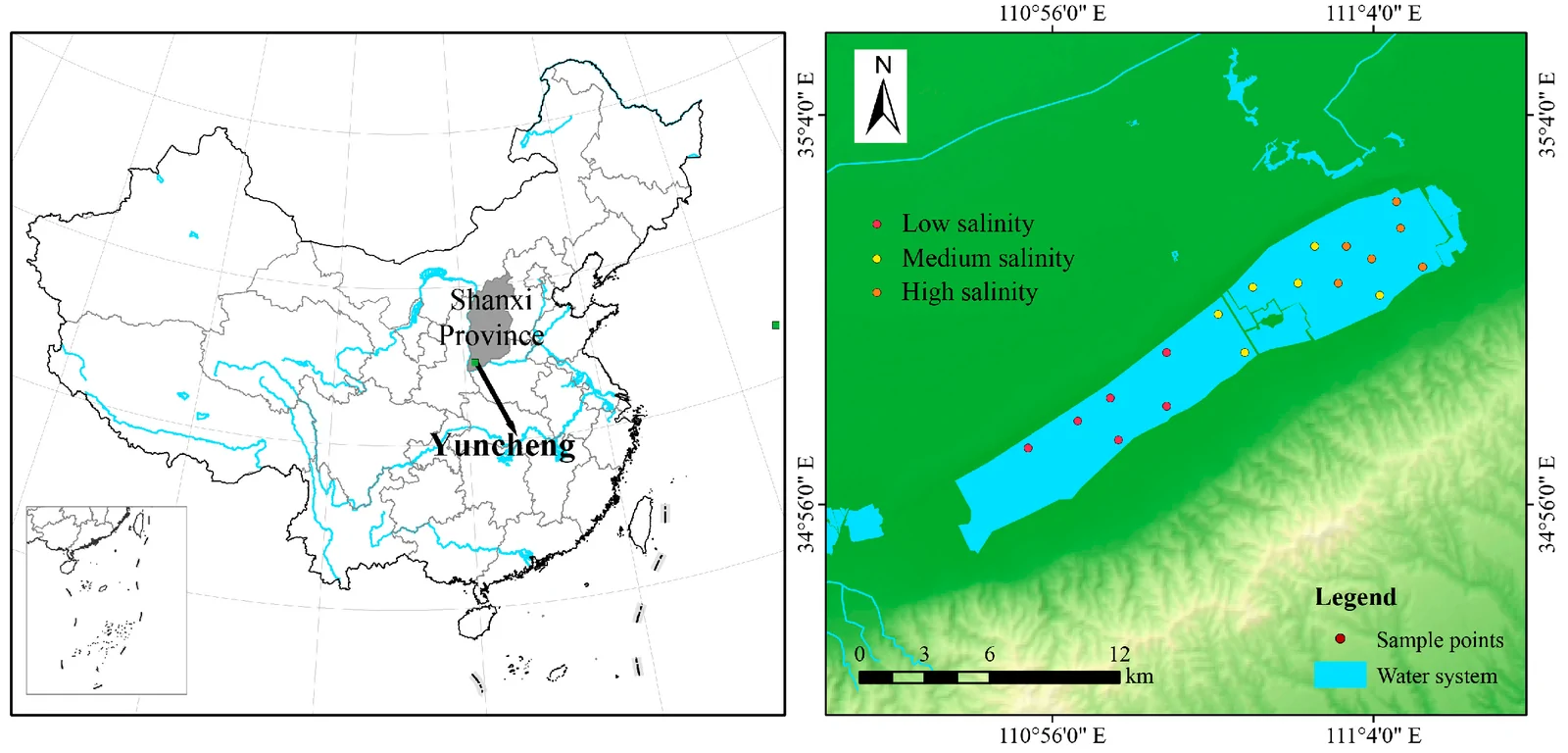
Location of the study area and the sampling points along the Yuncheng Salt Lake.

Yuncheng Salt Lake, located in southern Shanxi Province, China, is the world’s third-largest natural inland sodium sulfate lake. The lake is rich in sodium sulfate and other salts, which have led to increased soil salinization in the surrounding area. Shoreline vegetation is sparse and consists mainly of perennial herbaceous plants, such as reeds, and algae. The region has a mild, warm-temperate climate. All lake water samples were collected in October 2024 at the 18 sampling sites shown in Figure 1. A total of 54 samples were collected: three biological replicates at each of six sites per salinity category, yielding 18 samples for each category (low, medium, and high salinity). Surface water (0–0.5 m) was collected using sterile water samplers, and salinity and pH were measured on site. Water samples were filtered through sterile 0.22 μm filters to collect microorganisms; the filters were immediately flash-frozen in liquid nitrogen and stored at −80 °C for subsequent DNA extraction. Additional water samples were collected for laboratory physicochemical analysis. Dissolved organic carbon (DOC) was measured with a total organic carbon (TOC) analyzer by high-temperature catalytic combustion, with the produced CO_2_ quantified by non-dispersive infrared (NDIR) absorption. Total phosphorus (TP) was determined by potassium persulfate digestion followed by ammonium molybdate spectrophotometry, and total nitrogen (TN) by alkaline potassium persulfate digestion followed by UV spectrophotometry. Based on the observed salinity distribution, the sampling sites were classified into three salinity groups: low (salinity < 10%), medium (10% ≤ salinity < 20%), and high (salinity ≥ 20%) (Table A1). Salinity classification schemes for inland saline waters vary considerably among regions and research contexts [15]; the thresholds used here were therefore defined to reflect the natural salinity gradient of Yuncheng Salt Lake (Table A1) rather than a single universal classification.

### 2.2. Metagenomic Sequencing, Assembly, and Functional Annotation

Genomic DNA was extracted using the TIANMicrobe Magnetic Envir-DNA Kit 9 (Tiangen Biotech, Beijing, China), following the manufacturer’s instructions. DNA concentration and quality were assessed using a NanoDrop 2000 spectrophotometer (Thermo Fisher Scientific, Waltham, MA, USA) and 1% agarose gel electrophoresis, respectively, followed by paired-end sequencing on the Illumina HiSeq 4000 platform (Illumina Inc., San Diego, CA, USA). Raw reads were processed using fastp v0.23.1 to generate high-quality clean reads [16]. Metagenomic assembly was performed using MEGAHIT v1.1.2; contigs shorter than 300 bp were removed, and assembly quality was evaluated using QUAST v2.3 [17]. Coding sequences were predicted using MetaGeneMark v3.26. Redundant sequences were removed using MMseqs2 (v11-e1a1c) at 95% sequence identity and 90% coverage thresholds, yielding a non-redundant gene set for subsequent analyses [18].

The remaining high-quality reads were then aligned using SOAPaligner v2.21, and gene abundance was calculated as the number of reads divided by the relative gene length [19]. DIAMOND v0.8.35 was used to align the data against the NCBI-NR and KEGG databases for taxonomic and functional annotation [20], identifying functional genes involved in microbial carbon (C), nitrogen (N), and phosphorus (P) metabolism.

### 2.3. Statistical Analysis

All data analyses were primarily performed using R software (version 4.0.4). Principal Coordinate Analysis (PCoA) based on Bray–Curtis distance and ANOSIM were used to test for differences among the different salinity gradients. Redundancy Analysis (RDA) was used to assess the effects of environmental factors on carbon (C), nitrogen (N), and phosphorus (P) cycling processes in the salt lake water. The variance inflation factor threshold was set to 10 to reduce multicollinearity. The log2-fold change (LFC) across different salinity gradients was calculated using the apeglm shrinkage algorithm in DESeq2. Subsequently, the Wilcoxon rank-sum test (Mann–Whitney U test) was used to compare differences in gene abundance between different salinities (FDR-corrected *p* < 0.05) [21]. The Kruskal–Wallis test was used to determine statistically significant differences in microbial taxa (relative abundance > 0.1%) across different salinity levels. Associations between water physicochemical properties and microbial taxa/functional genes were assessed using the Mantel test in the “ggcor” package (R software version 4.0.4). Correlations between C, N, and P cycle genes and water physicochemical properties were presented as heatmaps. To construct a strong co-occurrence network, correlations were calculated only for functional genes with abundances greater than 1% [22], only correlations with |ρ| > 0.7 and FDR-corrected *p* < 0.05 were retained as network edges. Network visualization was performed using Gephi software (version 0.9.2) (https://gephi.org/ accessed on 15 March 2026).

## 3. Results

### 3.1. Effects of Salinity on Microbial Diversity and Functional Group Composition

**Figure 2:**
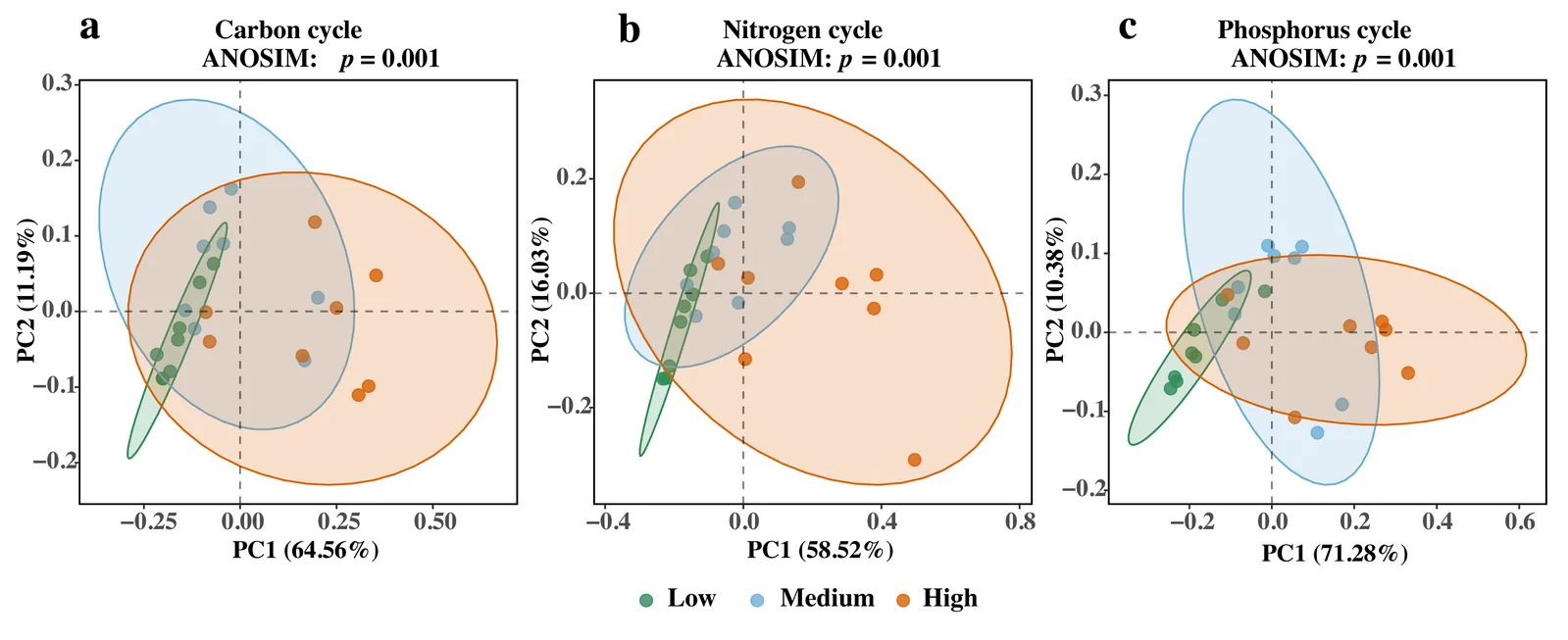
Microbial functional profiles of carbon (**a**), nitrogen (**b**), and phosphorus (**c**) cycles in salt lake waters, identified using principal coordinate analysis (PCoA) based on Bray–Curtis distance.

**Figure A1:**
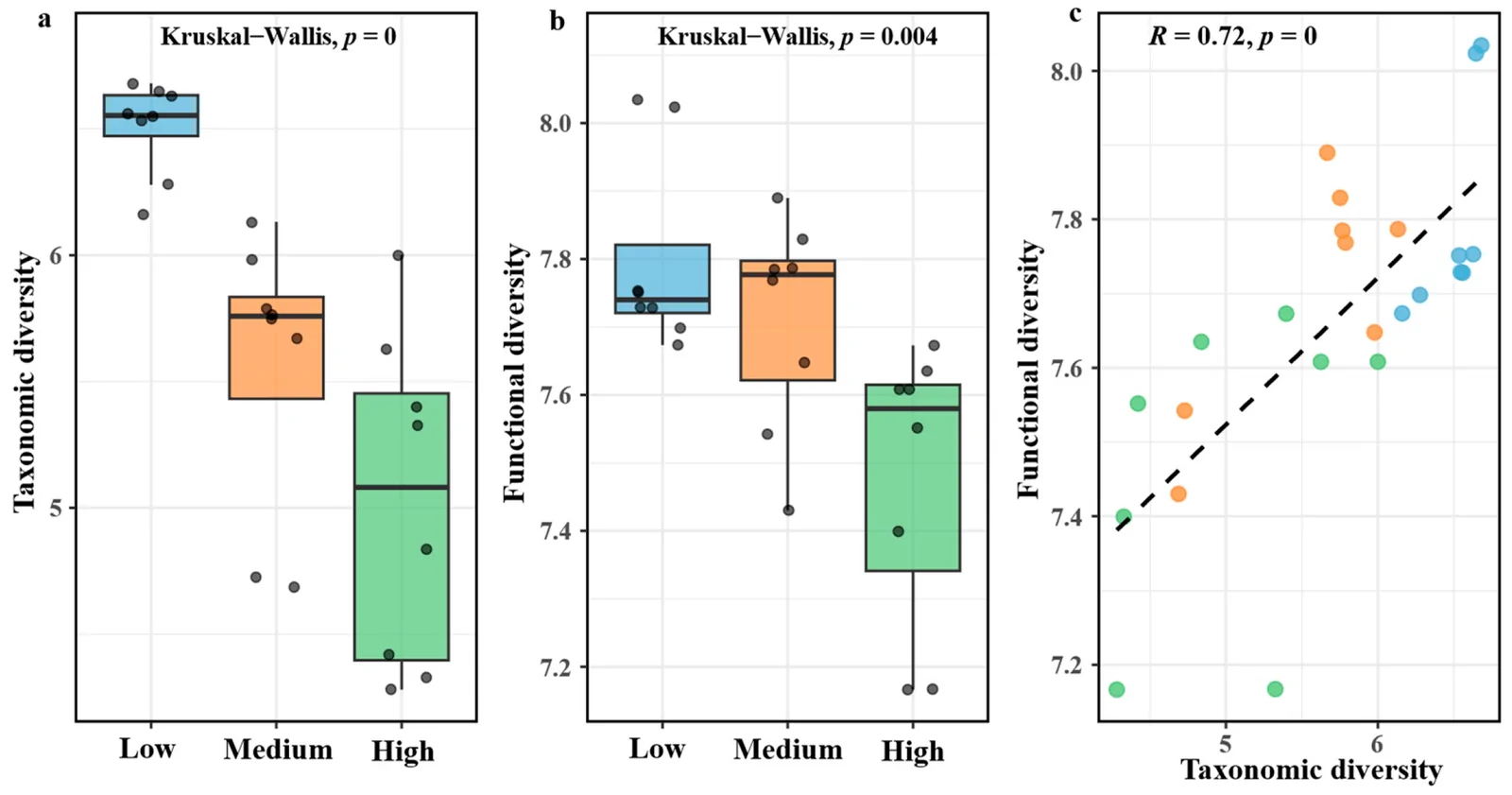
Boxplots showing taxonomic (**a**) and functional (**b**) alpha (Shannon) diversity under different salinity conditions. The scatter plot shows the correlation between functional diversity and taxonomic diversity (**c**).

**Figure A2:**
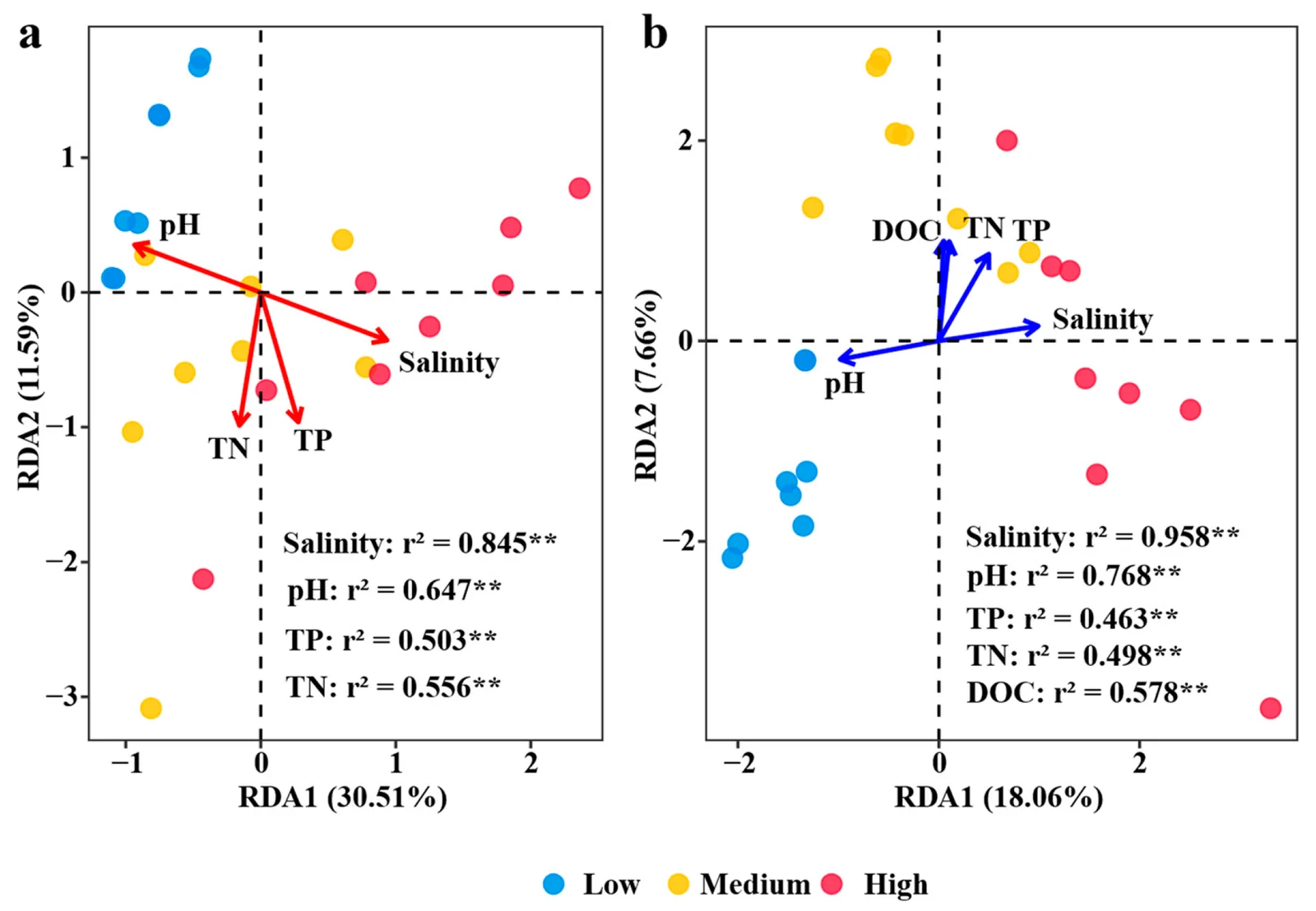
Redundancy analysis of microbial taxa (**a**) and functional genes (**b**) at the phylum and KO level associated with environmental factors, and only the significant correlations were shown. The asterisks indicate significance at probability levels equal to *p* < 0.01 (**).

The physicochemical parameters of the sampling sites are summarized in Table A1; diversity indices and redundancy analysis results are shown in Figure A1 and Figure A2, respectively. Both taxonomic and functional diversity, assessed by the Shannon index, decreased with increasing salinity (Figure A1), with maximum values of 6.68 and 8.03, respectively. Taxonomic and functional diversity were significantly positively correlated (*p* < 0.05) (Figure A1). RDA showed that both microbial taxa and functional genes were significantly correlated with environmental factors, including salinity (*r*^2^ = 0.845, *p* < 0.01; *r*^2^ = 0.958, *p* < 0.01), pH (*r*^2^ = 0.647, *p* < 0.01; *r*^2^ = 0.768, *p* < 0.01), total nitrogen (TN, *r*^2^ = 0.556, *p* < 0.01; *r*^2^ = 0.498, *p* < 0.01), and total phosphorus (TP, *r*^2^ = 0.503, *p* < 0.01; *r*^2^ = 0.463, *p* < 0.05) (Figure A2). PCoA confirmed significant differences (*p* < 0.05) in C, N, and P cycling gene profiles among the three salinity groups (Figure 2), indicating that microbial communities and C, N, and P cycling gene profiles differed among salinity groups in the salt lake.

### 3.2. Carbon Cycling Gene Profiles Differ Significantly Among Salinity Gradients

**Figure 3:**
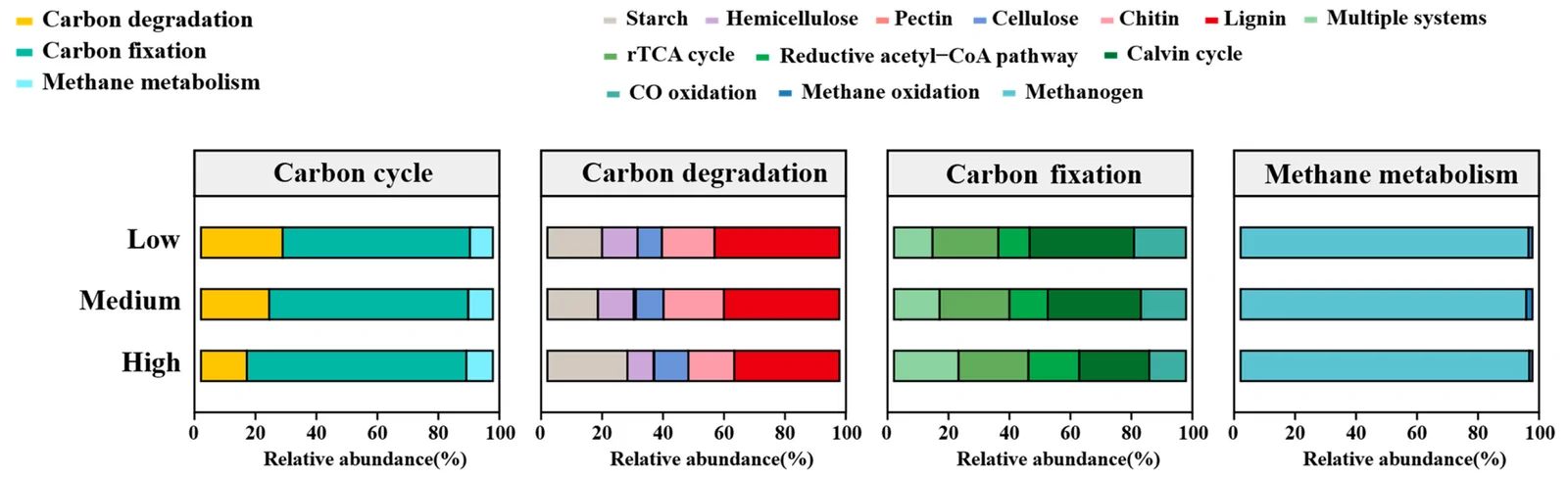
Stacked bar graph showing the relative abundances of carbon cycling genes under different salinity conditions.

Carbon cycling genes were classified into three categories: carbon fixation, carbon degradation, and methane metabolism (Table A2). Carbon fixation pathways primarily included multiple systems: the reductive tricarboxylic acid cycle (rTCA cycle), the reductive acetyl-CoA pathway, the Calvin cycle, and carbon monoxide (CO) oxidation. Among these, the Calvin cycle accounted for 24.77–35.90% of carbon fixation genes, the rTCA cycle for 22.47–24.17%, and the reductive acetyl-CoA pathway for 10.74–17.02%. The carbon degradation group is divided into labile carbon and recalcitrant carbon, namely starch, hemicellulose, pectin, cellulose, chitin, and lignin; among these, lignin-degradation genes showed the highest relative abundance (Figure 3).

**Figure 4:**
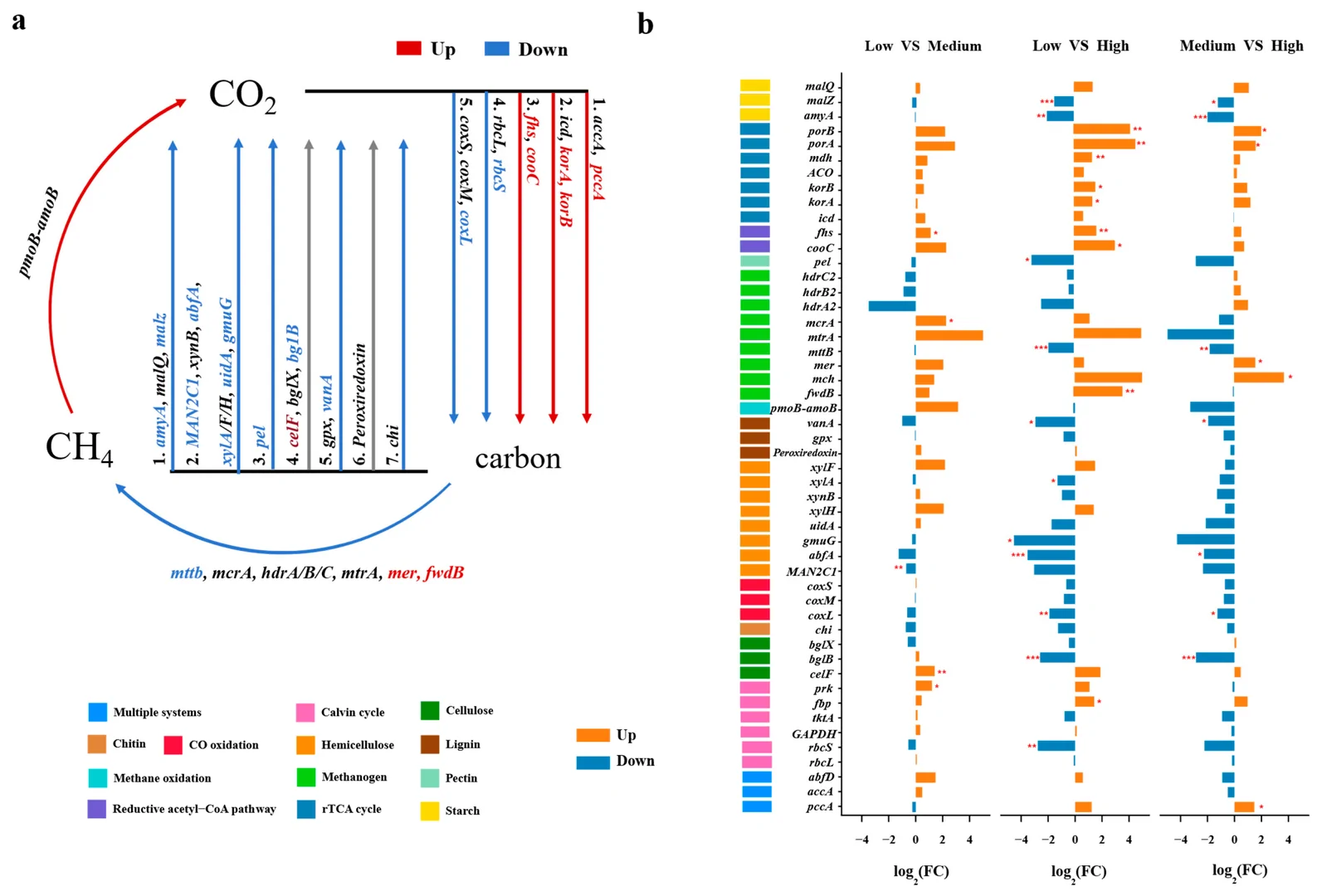
(**a**) Diagram depicting the different carbon cycling processes based on metagenomic sequencing. Orange indicates that gene abundance increases significantly with salinity, while blue indicates that gene abundance decreases significantly with salinity. (**b**) Bar charts reflect the fold change of functional genes between different salinities. Asterisks (*) indicate significant differences. Significance levels of each predictor are “*” *p* < 0.05, “**” *p* < 0.01, “***” *p* < 0.001.

Wilcoxon rank-sum tests showed that carbon cycling gene abundances differed significantly among salinity groups (Figure 4). In carbon fixation, *pccA*, *korA*, *korB*, *fhs*, and *cooC* abundances increased significantly with salinity, while those of *rbcS* and *coxL* decreased significantly. In carbon degradation, *amyA*, *malZ*, *MAN2C1*, *abfA*, *xylA*, *uidA*, *gmuG*, *pel*, *bglB*, and *vanA* abundances decreased significantly with salinity, while *celF* abundance increased significantly. In methane metabolism, *mer* and *fwdB* abundances were significantly higher in high-salinity waters, while *mttB* abundance was significantly reduced. These results indicate that carbon fixation, carbon degradation, and methane metabolism gene abundances were all associated with salinity.

**Figure A3:**
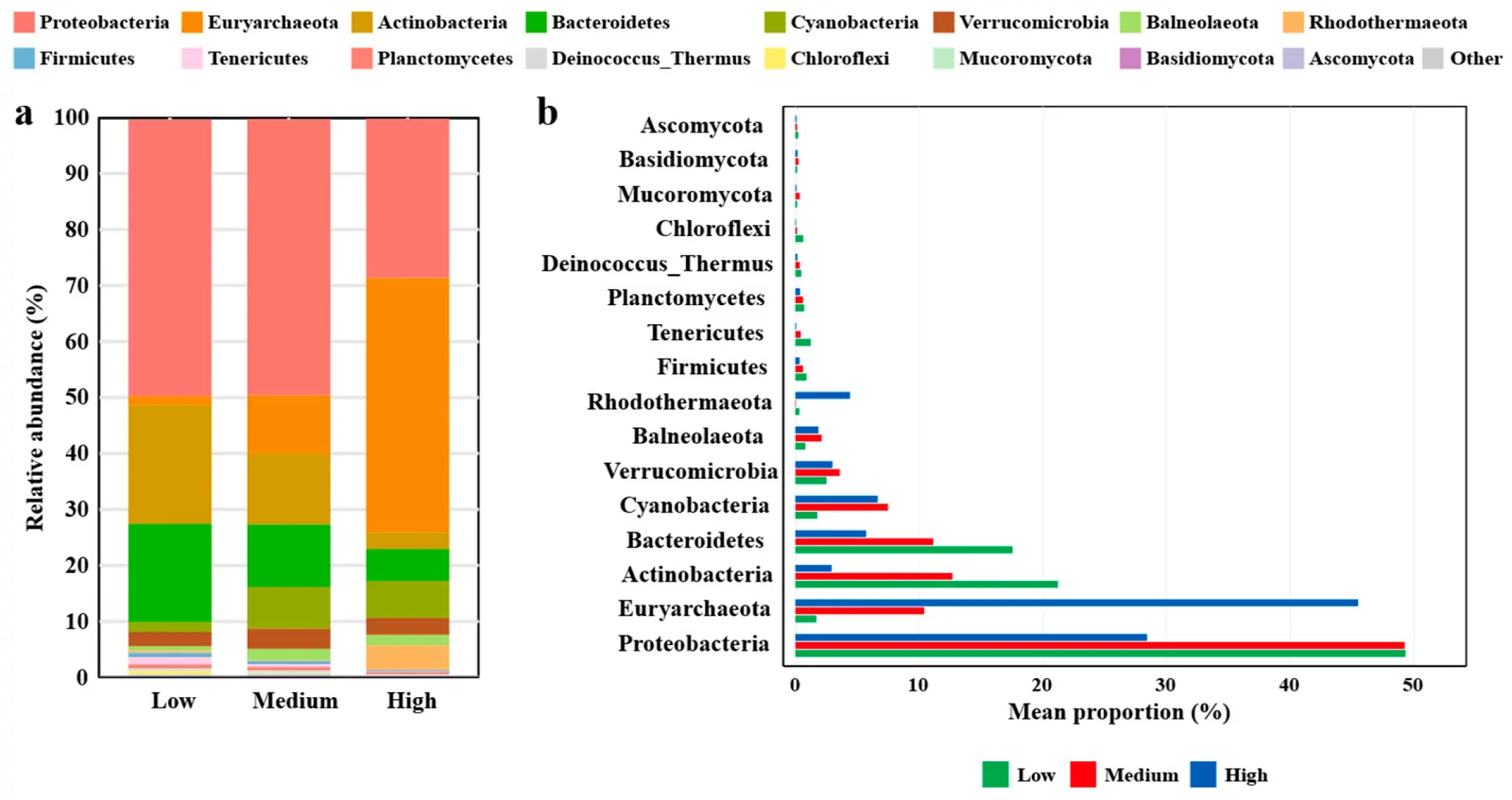
The relative abundance of microbial taxa by carbon-cycling genes at the phylum (**a**) and the comparison between different salinity (**b**).

Phylogenetic annotation of carbon cycle genes (Figure A3) indicates that Proteobacteria, Bacteroidetes, and Actinobacteria dominated low-salinity waters. As salinity increased, Cyanobacteria and Euryarchaeota abundances rose rapidly, while Proteobacteria and Bacteroidetes abundances continued to decline. Euryarchaeota became the dominant phylum in high-salinity waters.

### 3.3. Nitrogen Cycling Gene Profiles Differ Significantly Among Salinity Gradients

**Figure 5:**
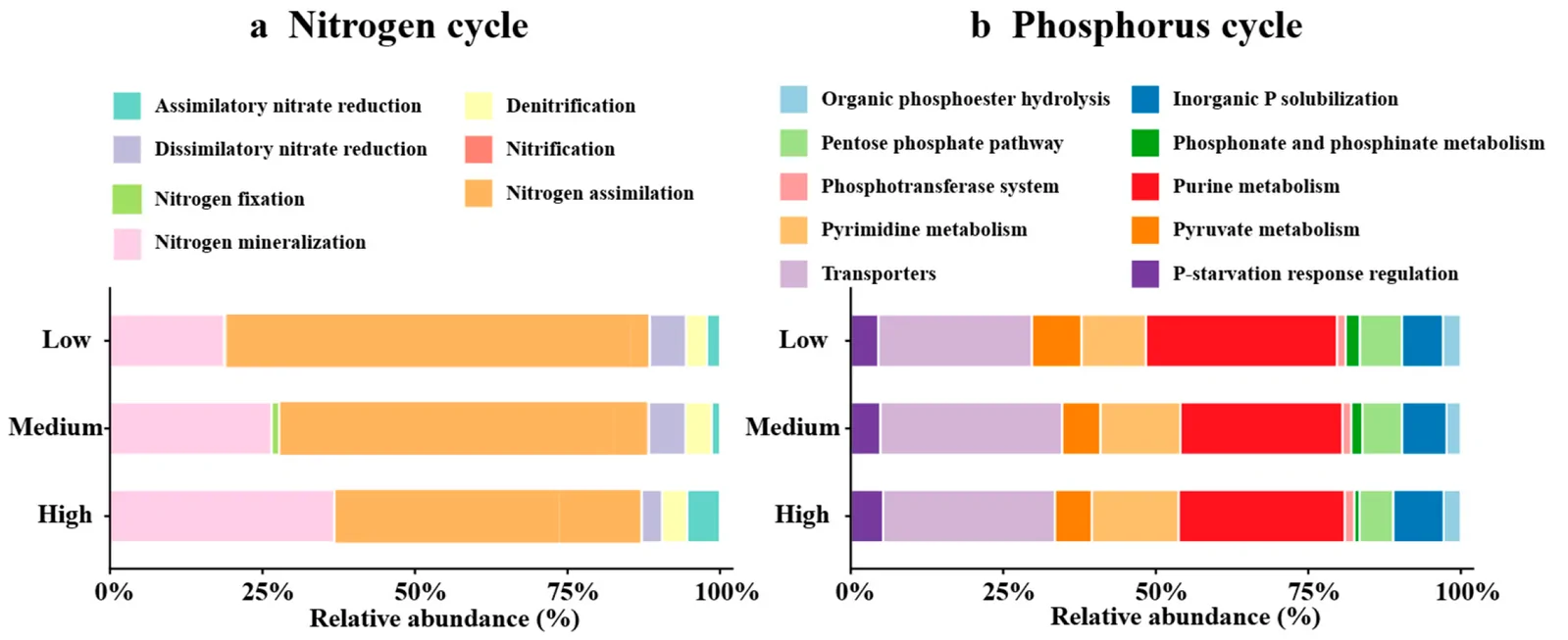
Stacked bar graph showing the relative abundances of nitrogen (**a**) and phosphorus (**b**) cycling genes under different salinity conditions.

**Figure 6:**
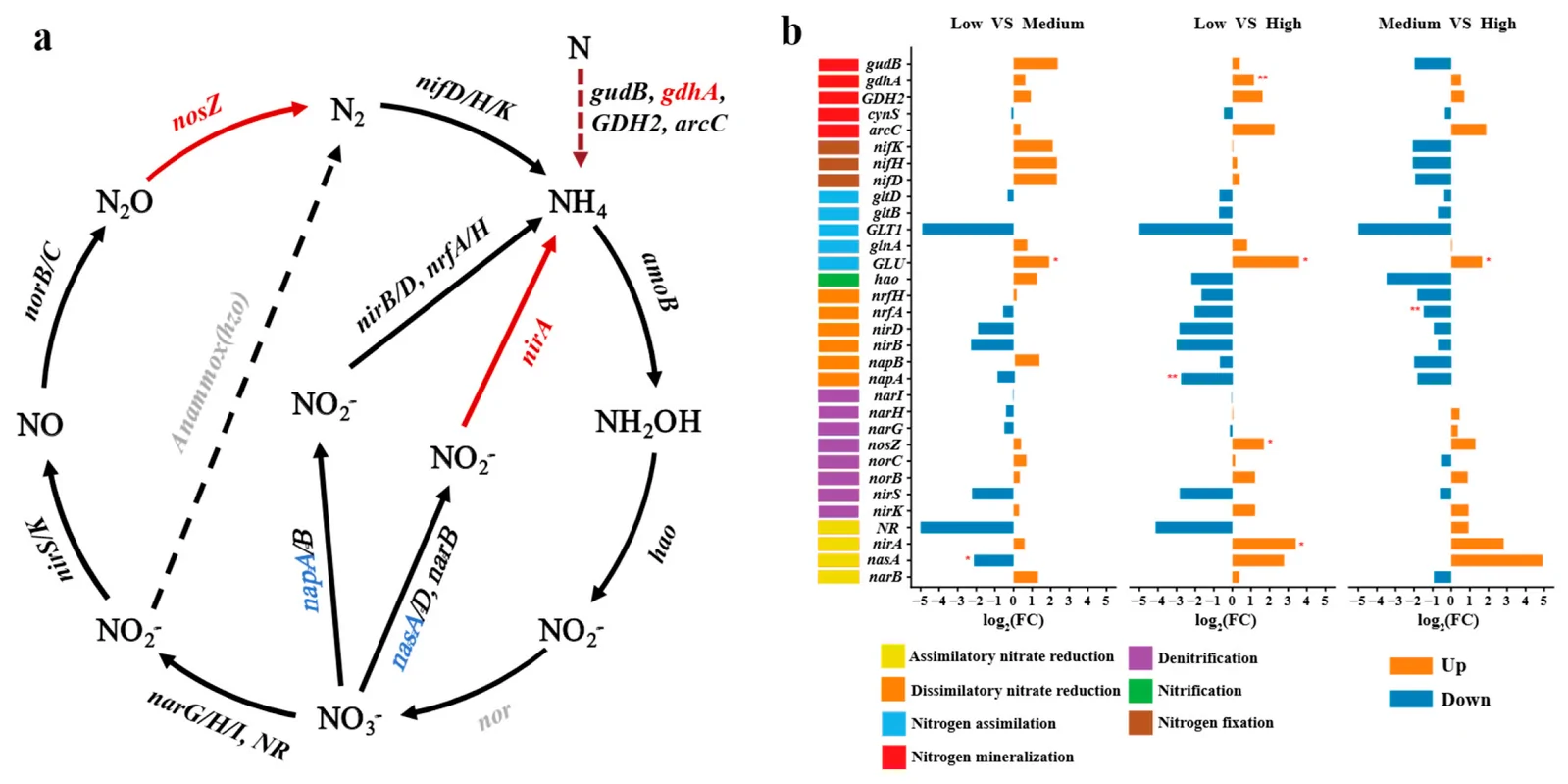
(**a**) Diagram depicting the different nitrogen cycling processes based on metagenomic sequencing. Orange indicates that gene abundance increases significantly with salinity, while blue indicates that gene abundance decreases significantly with salinity. (**b**) Bar charts reflect the fold change of functional genes between different salinities. Asterisks (*) indicate significant differences. Significance levels of each predictor are “*” *p* < 0.05, “**” *p* < 0.01.

The nitrogen cycle in Yuncheng Salt Lake primarily includes nitrogen fixation, nitrification, denitrification, assimilatory nitrate reduction (ANRA), dissimilatory nitrate reduction (DNRA), nitrogen mineralization, and nitrogen assimilation. Among these, nitrogen assimilation genes showed the highest relative abundance, accounting for 36.62–66.24% (Figure 5). Salinity affected individual steps of the nitrogen cycle differently. Nitrogen mineralization (*gdhA*), nitrogen assimilation (*GLU*), and ANRA (conversion of NO_2_ to NH_4_, *nirA*) showed significantly higher abundances as salinity increased. As salinity increased, the *napA* (NO_3_^−^ → NO_2_^−^) abundances decreased, *nirK* (NO_2_^−^ → NO) and *norB* (NO → N_2_O) abundances showed an upward trend but did not reach statistical significance; *nosZ* (N_2_O → N_2_) abundances increased significantly (Figure 6).

**Figure A4:**
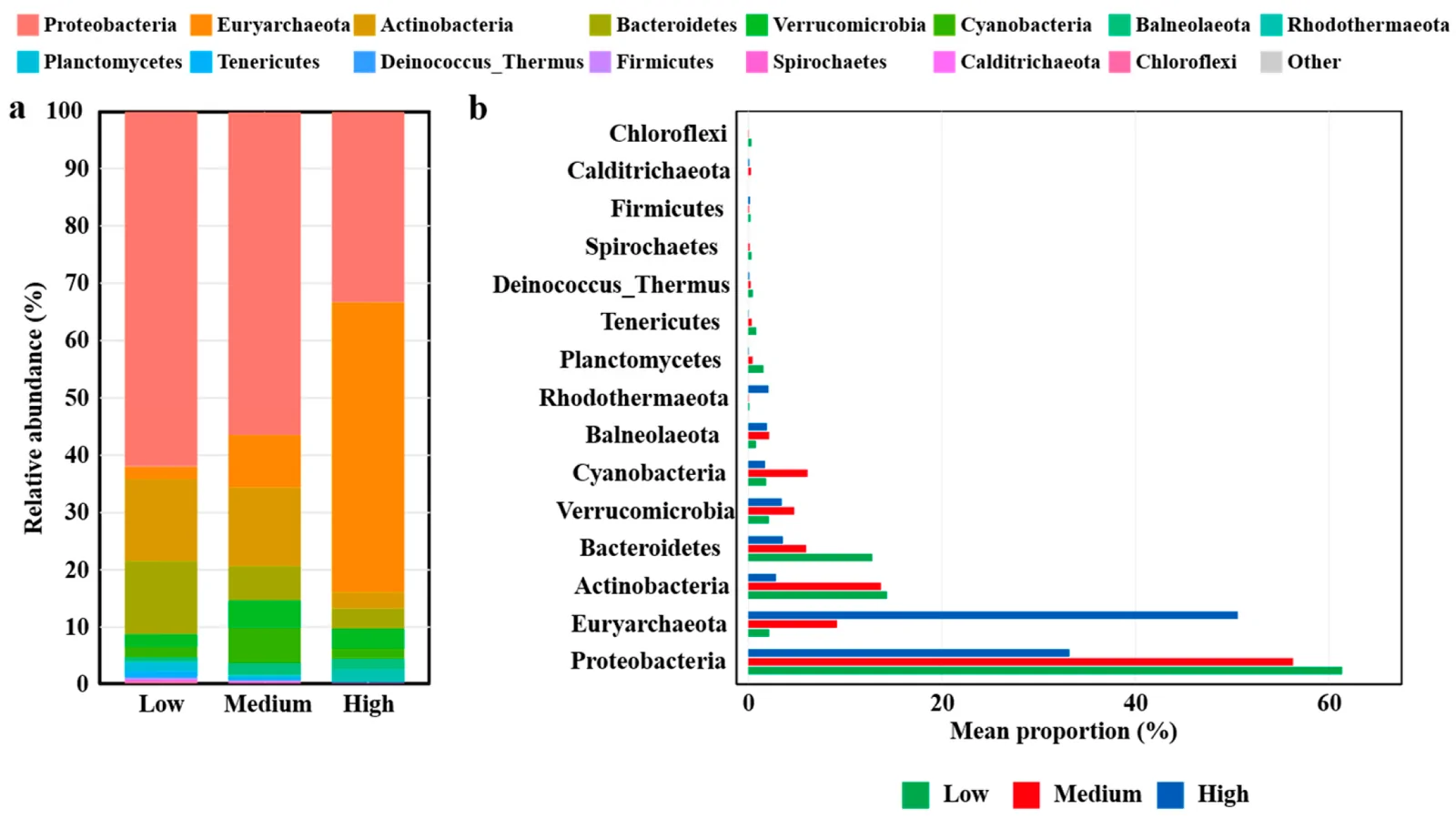
The relative abundance of microbial taxa by nitrogen-cycling genes at the phylum (**a**) and the comparison between different salinities (**b**).

In low-salinity waters, bacteria dominated all nitrogen cycling processes, and Proteobacteria was the key contributor, accompanied by Bacteroidetes, Actinobacteria, and Verrucomicrobia. In medium-salinity waters, the community entered a transitional phase: Proteobacteria abundance continued to decline, whereas Cyanobacteria and Balneolaeota abundances increased. In high-salinity waters, extremophilic halophilic archaea became the key drivers of nitrogen cycling (Figure A4).

### 3.4. Phosphorus Cycling Gene Profiles Differ Significantly Among Salinity Gradients

Phosphorus cycling processes primarily include organic phosphoester hydrolysis, inorganic P solubilization, the pentose phosphate pathway, phosphonate and phosphinate metabolism, the phosphotransferase system (PTS), purine metabolism, pyrimidine metabolism, pyruvate metabolism, transporters, and P-starvation response regulation. Among all phosphorus cycling processes, phosphorus transport and purine metabolism showed the highest relative abundances, accounting for 25.15–29.80% and 26.56–31.30%, respectively (Figure 5b).

**Figure 7:**
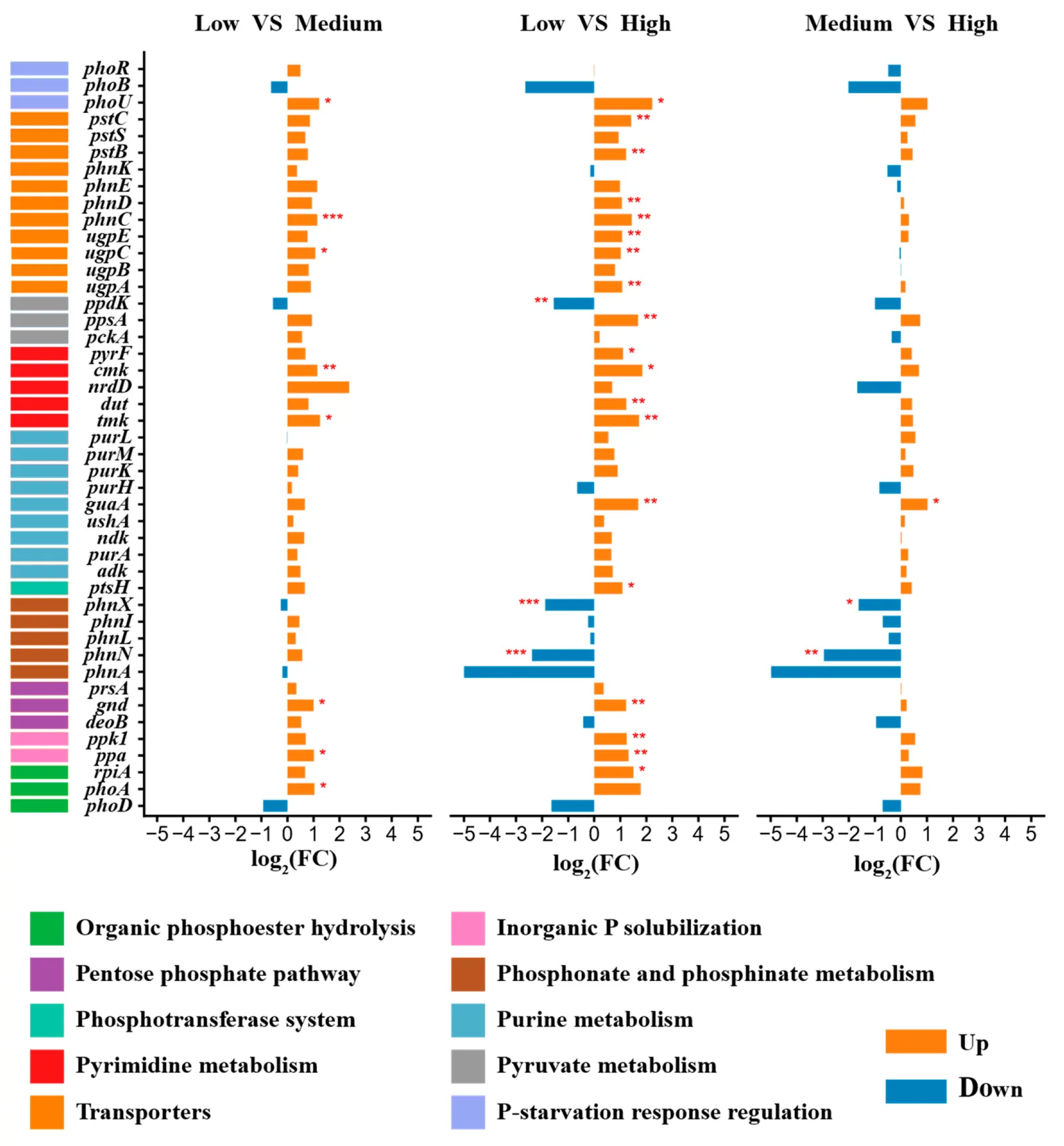
The bar chart shows the fold changes in the abundance of functional genes involved in the phosphorus cycle under different salinity conditions. Asterisks (*) indicate significant differences. Significance levels of each predictor are “*” *p* < 0.05, “**” *p* < 0.01, “***” *p* < 0.001.

Pyrimidine metabolism (*tmk*, *dut*, *cmk*), purine metabolism (*guaA*), transporters (*phnC/D/E*, *pstB/C*, *ugpA/E/C*), P-starvation response regulation (*phoU*), inorganic P solubilization (*ppa* and *ppk1*), and pyruvate metabolism (*ppsA*) gene abundances all increased significantly along the salinity gradient. *phnN/X* (phosphonate and phosphinate metabolism) abundances decreased significantly as salinity increased (Figure 7).

**Figure A5:**
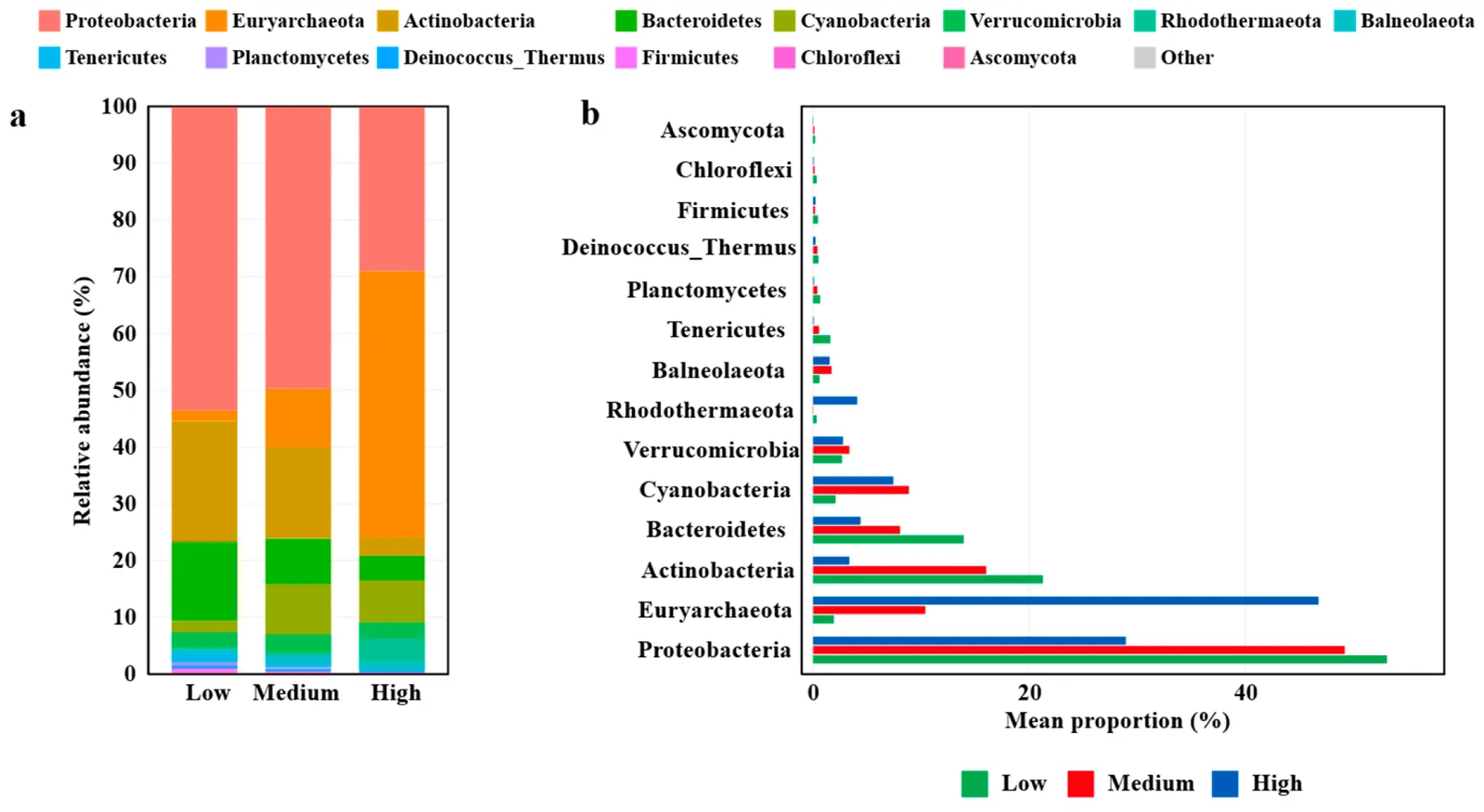
The relative abundance of microbial taxa by phosphorus-cycling genes at the phylum (**a**) and the comparison between different salinities (**b**).

Across the salinity gradient, Proteobacteria, Actinobacteria, and Bacteroidetes abundances gradually decreased, whereas Euryarchaeota and Cyanobacteria abundances gradually increased (Figure A5).

### 3.5. Relationship Between Microbial Functional Genes and Environmental Nutritional Factors

**Figure 8:**
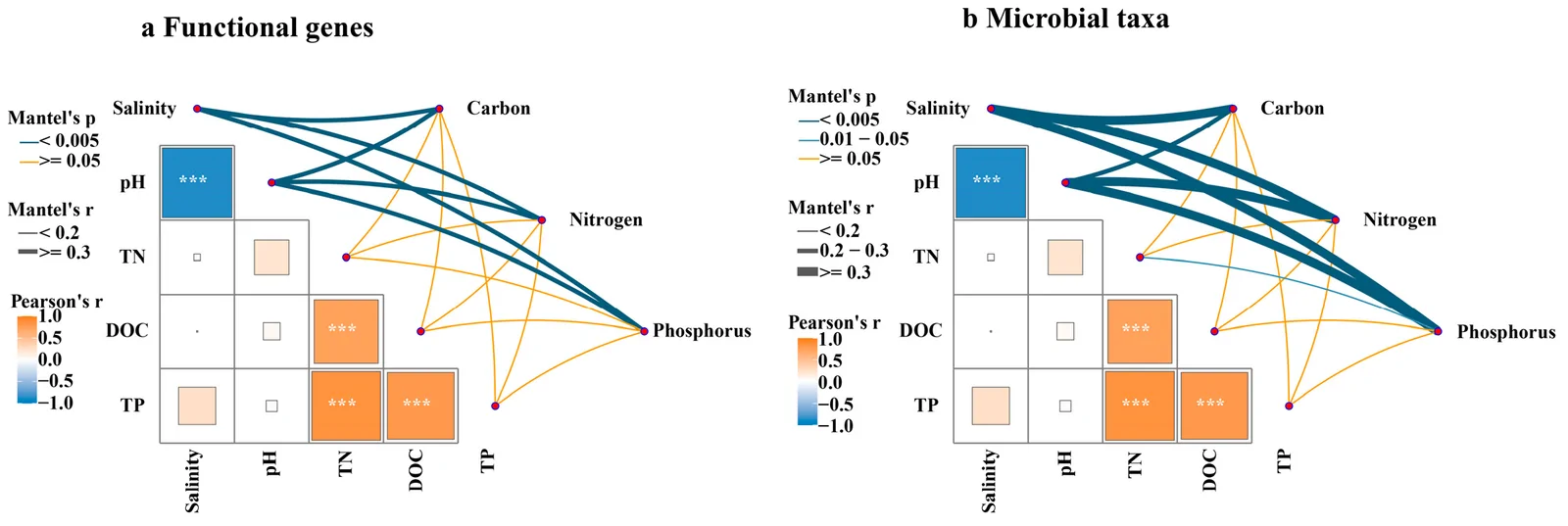
Associations between environmental factors and microbial functional genes and taxa. Pairwise correlations among environmental factors are shown with a colour gradient (Pearson’s r). Mantel tests assessed the relationships of carbon, nitrogen, and phosphorus cycling genes and microbial taxa with each environmental factor; edge width represents Mantel’s r, and edge colour indicates statistical significance. Asterisks (*) indicate significant differences, “***” *p* < 0.001.

**Figure A6:**
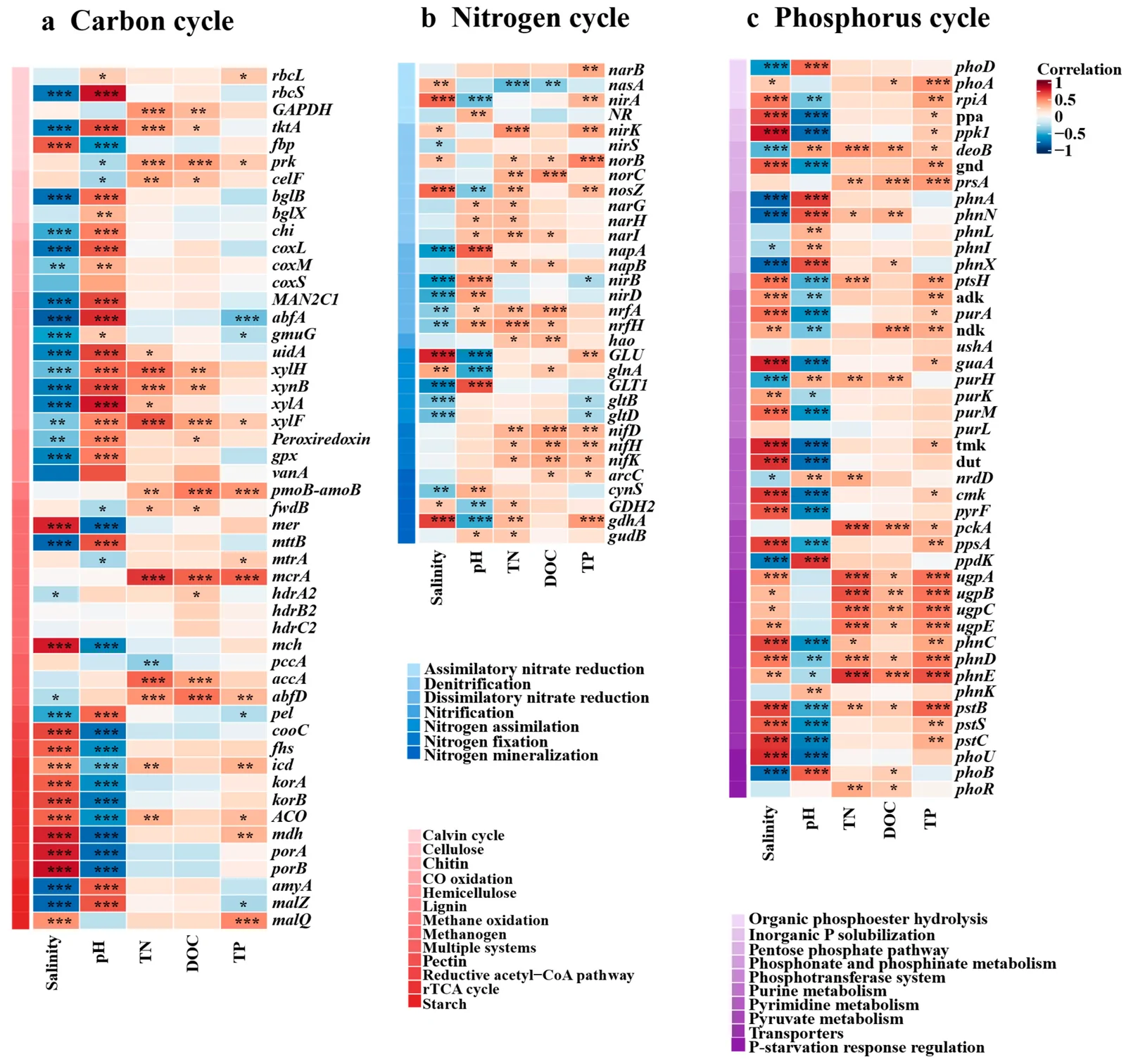
Heatmap analysis of the relationships between environmental factors and the abundance of carbon, nitrogen and phosphorus cycling genes. Significance levels of each predictor are “*” *p* < 0.05, “**” *p* < 0.01, “***” *p* < 0.001.

Mantel tests evaluated the relationships between C, N, and P cycling genes and physicochemical properties. Both salinity and pH significantly influenced the functional profiles of C, N, and P cycling as well as specific functional genes (Figure 8). To test whether salinity acted as the primary factor and whether it acted jointly with other environmental variables, we performed variance partitioning analysis (VPA) on the functional gene matrix and the microbial phylum matrix using the R package vegan (version 4.0.4). For functional genes, the full model explained 54.19% of the adjusted variation; salinity uniquely contributed 7.28%, whereas pH and the nutrient variables (TN, DOC, TP) together contributed 14.23% independently, with substantial shared effects. For microbial phyla, the full model explained 63.81%; salinity contributed 9.13% uniquely, while the nutrient group contributed 16.37% uniquely, and the shared fraction with salinity was large (36.85% in the two-group partition). These results indicate that salinity and other environmental factors act in concert. Heatmap analysis showed that salinity was significantly positively correlated with the rTCA and reductive acetyl-CoA pathways, particularly with *fhs*, *cooC*, *porA/B*, *icd*, *korA/B*, and *mdh*. Hemicellulose degradation genes (*xylA/F/H*, *xynB*) were significantly positively correlated with pH, DOC, and TN. Nitrogen fixation genes *nifD/H/K* were significantly positively correlated with multiple environmental factors such as TN, TP, and DOC, while the *gltB/D* were significantly negatively correlated with salinity and TP. Several phosphorus cycle processes, such as transporters, pyrimidine metabolism, and purine metabolism, showed significant positive correlations with salinity, carbon, nitrogen, and phosphorus, and significant negative correlations with pH (Figure A6).

**Figure 9:**
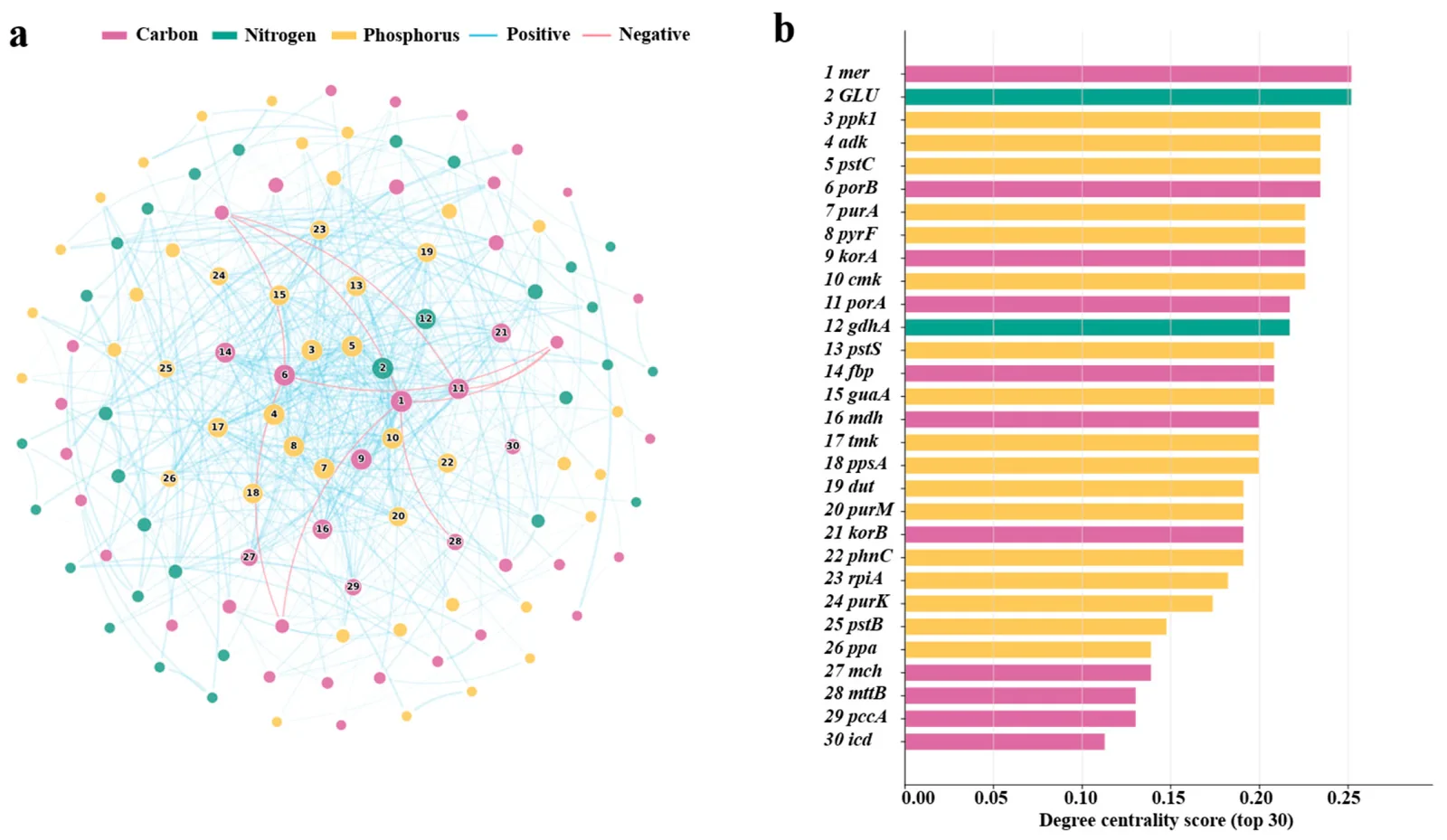
Network analysis between genes related to carbon, nitrogen and phosphorus cycling genes (**a**) and degree centrality scores for the networks (top 30) (**b**).

**Figure A7:**
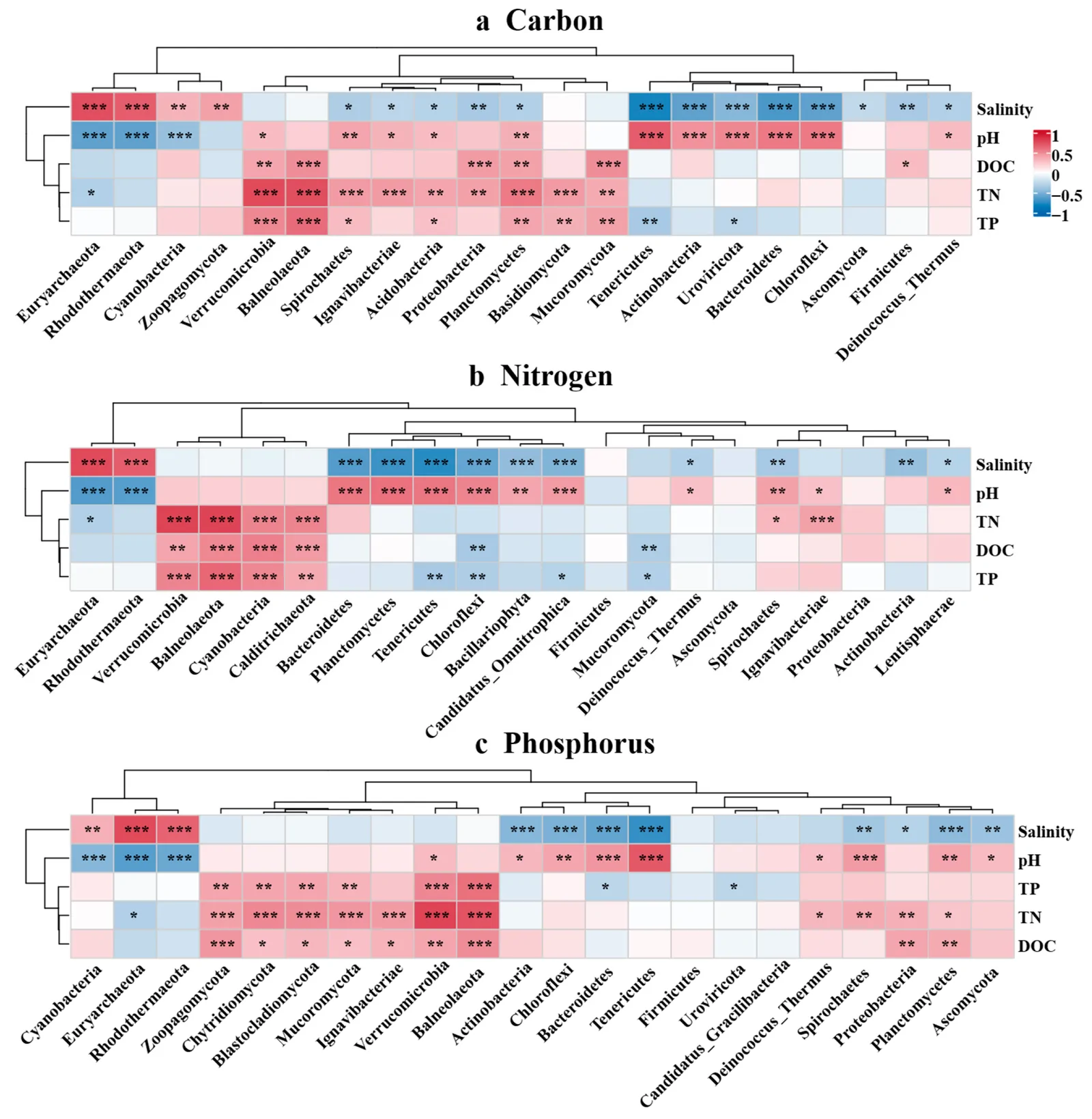
Heatmap analysis of the relationships between environmental factors and the relative abundance of microbial taxa of carbon, nitrogen and phosphorus cycling genes. Significance levels of each predictor are “*” *p* < 0.05, “**” *p* < 0.01, “***” *p* < 0.001.

Euryarchaeota and Rhodothermaeota showed significant positive correlations with salinity, indicating that these two halophilic groups are highly adapted to the high-salinity environment of salt lakes, and that higher salinity is associated with their growth (Figure A7). Verrucomicrobia, Balneolaeota, Proteobacteria, and Mucoromycota all showed significant positive correlations with DOC, TN, and TP, suggesting that these groups may benefit from higher nutrient availability. The co-occurrence network revealed dense positive co-occurrence associations among genes involved in carbon metabolism, nitrogen assimilation, and phosphorus metabolism in salt lakes (Figure 9a). Genes were ranked by degree centrality, and the degree values of genes ranked 1–30 were substantially higher than those of genes ranked below 30, which declined sharply toward the network average; the top 30 genes were therefore retained as the core highly connected hub nodes. The top 30 genes with the highest centrality included 11 carbon cycle genes (*mer*, *porB*, *korA*, *porA*, *fbp*, *mdh*, *korB*, *mch*, *mttB*, *pccA*, *icd*), 2 nitrogen cycle genes (*GLU* and *gdhA*), and 17 phosphorus cycle genes (*ppk1*, *pstC*, *pyrF*, *purA*, *cmk*, *guaA*, *tmk*, *ppsA*, *purM*, *pstS*, *dut*, *phnC*, *rpiA*, *pstB*, *adk*, *ppa*, and *purK*) (Figure 9b). Spearman’s correlation analysis revealed complex interrelationships among carbon, nitrogen, and phosphorus cycling genes. Among these highly central genes, *mer* (5,10-methylenetetrahydromethanopterin reductase) showed a significant positive correlation with carbon cycle genes (e.g., *porA/B*), nitrogen cycle genes (*GLU*, *gdhA*), and phosphorus cycle genes (*guaA*, *ppk1*, *cmk*).

## 4. Discussion

### 4.1. Associations Between Salinity and Microbial Functional Genes Involved in Carbon, Nitrogen, and Phosphorus Cycles

#### 4.1.1. Responses of the Carbon Cycle to Salinity

Carbon fixation, carbon degradation, and methane metabolism are the three major processes through which microorganisms obtain energy and nutrients [23]. In this study, carbon fixation genes were the most abundant functional category in the Yuncheng Salt Lake, and their abundances were significantly higher at higher salinity. However, different carbon fixation pathways responded differently to salinity. As salinity increased, the relative contribution of Calvin cycle genes decreased, whereas the relative contributions of multiple systems, the acetyl-CoA pathway, and the rTCA cycle increased. Furthermore, carbon degradation gene abundances (chitin and lignin degradation) decreased at higher salinity. The low energy demand of the rTCA cycle (2 ATP per fixed carbon) may partly explain its increased contribution in high-salinity waters [24,25]. In terms of microbial contributions, rTCA cycle-related genes were primarily contributed by the archaea *Halalkalicoccus*, *Haloquadratum* (*korB*), *Halopenitus* (*mdh*), *Halobellus* (*korA*), and the cyanobacterium *Euhalothece*. In addition, the primary contributing group to the Calvin cycle is the archaea *Halobellus*, *Halopelagius*, *Halorubrum*, *Halostella*, *Haloplanus*, and *Halegenticoccus*. The higher carbon fixation potential provides the building blocks for halophilic microorganisms to synthesize carbon skeletons and metabolic intermediates (including osmoregulatory substances such as glycine and betaine), playing a crucial role in maintaining the carbon cycle in high-salinity ecosystems.

Organic carbon degradation typically reflects the consumption characteristics of an ecosystem [26]. A distinctive feature of carbon metabolism in the high-salinity environment examined in this study is that carbon degradation gene abundances were lower while carbon fixation gene abundances were higher. Furthermore, methane production gene abundances (*mttB*) decreased at higher salinity. Previously, Yang et al. (2022) [6] reported a weak positive correlation between methane metabolic pathways and salinity. In carbon degradation, hemicellulose-degrading genes decreased with increasing salinity. Hemicellulose degradation is primarily involved in *Nitriliruptor* and *Pontimonas* in Actinobacteria, Lewinella in Bacteroidetes, and *Nioella* (*xylH*) and *Salibaculum* (*xylF*) in Proteobacteria. Although archaea are the most abundant in high-salinity waters, it is not archaea but bacterial groups that contribute the most to hemicellulose degradation. This phenomenon suggests that in high-salinity ecosystems, there is not always a correlation between the abundance of microbial communities and their functional contributions; although archaea are numerically dominant, their functional role in hemicellulose degradation is relatively limited, whereas bacteria (particularly specific groups within the Actinobacteria, Bacteroidetes, Proteobacteria, and Rhodothermaeota) perform the primary degradation functions. In contrast, the degradation of lignin and chitin may depend on microbial groups or enzyme systems that are more sensitive to salinity and are therefore inhibited under high-salinity conditions.

#### 4.1.2. Responses of the Nitrogen Cycle to Salinity

Nitrogen is essential for the microbiome to withstand environmental stress [27]. In this study, salinity was associated with varying abundances of genes in different nitrogen cycling processes. The higher abundances of nitrogen mineralization genes (*gdhA*, *GDH2*, *arcC*) suggest that high salinity is associated with greater potential for conversion of organic nitrogen to inorganic nitrogen. In terms of species contributions, the nitrogen mineralization gene *gdhA* is primarily derived from the archaeon *Salinarchaeum*, Verrucomicrobia *Coraliomargarita*, and Rhodothermaeota *Salinivenus*. The gene *nirA* encodes an assimilatory nitrite reductase that catalyzes the reduction of NO_2_^−^ to NH_4_^+^; its higher abundance suggests that microorganisms may be increasingly using nitrate-derived nitrogen under high-salinity conditions. The co-occurrence of higher *nirA* with *glnA* and *GLU* abundances suggests a potential statistical association between nitrate reduction genes and glutamine/glutamate synthesis genes [28,29]. Genes related to nitrogen assimilation are primarily contributed by halophilic archaea. *glnA* is predominantly sourced from the archaea *Halobellus*, *Natronomonas*, *Halogeometricum*, and *Salinarchaeum*, as well as the halotolerant cyanobacterium *Euhalothece*; *GLU* is predominantly sourced from the archaea *Halobellus* and *Salinarchaeum*; *gltB* (glutamate synthase large subunit) is derived from the Actinobacteria *Pontimonas* and the Proteobacteria *Spiribacter*; *gltD* is contributed by the Proteobacteria *Spiribacter*. The abundance of nitrogen fixation-related genes (*nifK*, *nifH*, *nifD*) showed an initial increase followed by a decline, peaking in medium-salinity waters and decreasing again at high salinity. This is related to changes in the composition of microbial communities in waters of different salinities; for example, the abundance of nitrogen-fixing cyanobacteria was highest in moderately saline waters.

Genes associated with denitrification (*nirK*, *norB*, *nosZ*) showed higher abundances at higher salinity. This difference may stem from: (i) oxygen concentrations in salt lake waters are typically low, providing a more favorable anaerobic microenvironment for denitrification. Li et al. (2021) [11] also noted that high-salinity environments are typically associated with lower oxygen concentrations, and denitrifying microorganisms require anaerobic conditions for denitrification; (ii) the species diversity of denitrification-related genes in salt lakes is relatively broad, encompassing archaea (*Halorubrum*, *Halobellus*, *Halovenus*), Proteobacteria (*Roseovarius*, *Salinihabitans*), Bacteroidetes (*Salinibacter*), and Balneolaeota (*Rhodohalobacter*, Gracilimonas). This broad distribution suggests that denitrification in high-salinity environments is jointly maintained by multiple halotolerant groups. *nosZ* encodes nitrous oxide reductase, which catalyzes the reduction of N_2_O to N_2_. This is the only key step in the denitrification pathway capable of reducing greenhouse gas emissions. Its significant positive correlation with salinity suggests that as salinity increases, the ability of microorganisms to convert N_2_O to N_2_ also increases. Research by Sun et al. (2025) [30] indicated that DOC can activate heterotrophic nitrification and denitrification processes by providing substrates and energy under high-salinity conditions, thereby significantly promoting N_2_O production. *nirK* (nitrite reductase) is sourced from *Roseovarius* (Proteobacteria), *Gracilimonas* (Balneolaeota), and the archaea *Halobellus* and *Halovenus*; *norB* (nitric oxide reductase) is sourced from the archaea *Halorubrum* and *Halobellus*, and *Roseovarius* (Proteobacteria). This study found that the abundances of genes associated with the dissimilatory nitrate reduction to ammonium (DNRA) process were significantly lower with increasing salinity, while those associated with assimilatory nitrate reduction (ANRA) showed a trend toward enhancement. These results are consistent with the findings of Li et al. (2024) [10].

#### 4.1.3. Responses of the Phosphorus Cycle to Salinity

At higher salinity, transporter and pyrimidine metabolism gene abundances were higher, while the proportion of purine metabolism in the total abundance of phosphorus cycling genes showed a downward trend. In high-salinity environments, microorganisms face extreme osmotic stress, leading to a surge in energy demand to maintain ion homeostasis and biosynthetic activity. Purine metabolism comprises the synthesis and degradation of purine nucleotides and also provides energy for cellular metabolism; it may enable certain bacteria and fungi to hydrolyze organic phosphorus into inorganic phosphate, thereby promoting phosphorus uptake by primary producers [31]. Regarding transporters, archaea such as *Salinigranum*, *Halopelagius*, *Haloplanus*, *Natrialba*, and *Haloferax* carried the majority of genes encoding high-affinity phosphate transport systems (*pst* and *phn* families). In terms of nucleotide metabolism, archaea such as *Halogeometricum*, *Haloplanus*, and *Halorubrum* dominated the contribution to pyrimidine metabolism genes, while purine metabolism was primarily contributed by *Euhalothece*, *Halorubrum*, *Natronoarchaeum*, *Salinarchaeum*, and other halophilic archaea. In addition, *Euhalothece* (Cyanobacteria) and *Spiribacter* (Proteobacteria) also participate in phosphate transport (*phnC*, *pstC*, *pstS*) and purine metabolism (*purM*). This reflects the differentiation in salt stress response strategies among different microbial groups, revealing a community response pattern dominated by halophilic archaea and supplemented by Cyanobacteria and Proteobacteria.

### 4.2. Functional Gene Networks of the Carbon, Nitrogen, and Phosphorus Cycles

The complexity and topological characteristics of co-occurrence networks provide insights into the relationships among functional genes [32], and network analysis can reveal how the co-abundance patterns of functional genes respond to environmental changes [33]. Interactions among microorganisms shape their functional diversity, and previous studies have shown that changes in microbial network structure can affect ecosystem function [34,35,36]. In the studied salt lake, positive co-occurrence associations predominated, indicating that the abundance patterns of most C, N, and P cycling genes were not independent of one another along the salinity gradient. Among the top 30 genes with the highest degree centrality, carbon cycle genes (11), phosphorus cycle genes (17), and nitrogen cycle genes (2) formed a highly interconnected functional network. mer (5,10-methylenetetrahydromethanopterin reductase) was significantly positively correlated with carbon fixation genes (porA/B) and nitrogen mineralization genes (gdhA), indicating a statistical association among methanogenesis-related, carbon-fixation-related, and nitrogen-mineralization-related gene abundances. Some methanogenic archaea couple carbon fixation with methane production and exhibit high metabolic activity [37].

This cross-elemental coupling indicates that C, N, and P cycles in salt lake ecosystems do not operate independently but are closely intertwined through microbial networks. Multifunctional microbial groups such as Proteobacteria and Verrucomicrobia were significantly positively correlated with DOC, TN, and TP across all three cycles, corroborating this coupling at the species level. Previous studies have shown that, in high-salinity environments, the carbon and phosphorus cycles are relatively complex and provide more opportunities for interactions between the two cycles. Compared with simple co-occurrence networks, complex networks support more efficient transfer of resources and information, promoting greater functionality [38]. Furthermore, highly complex networks may lead to more stable microbial communities and greater tolerance to environmental disturbance, because complex communities contain more microbial species, which facilitates faster and more complete decomposition of organic matter and phosphorus [39]. In this study, carbon cycle genes (11) and phosphorus cycle genes (17) dominated the network, and the numerous significant associations between them support the view that carbon and phosphorus cycles are highly coupled in high-salinity environments and that functional networks tend to be complex. Network analysis further identified mer, ppk1, and other genes as hub nodes linking the carbon and phosphorus cycles, which may play a key role in maintaining the stability and resilience of element cycling in salt lake ecosystems.

## 5. Conclusions

This study used the natural salinity gradient in Yuncheng Salt Lake as its research system and employed metagenomic methods to reveal associations between salinity and microbial community structure and functional genes involved in carbon, nitrogen, and phosphorus cycles. Both microbial taxonomic and functional diversity decreased significantly with increasing salinity, and the two were significantly positively correlated (*p* < 0.05). The microbial community evolved from one dominated by the phyla Proteobacteria, Bacteroidetes, and Actinobacteria at low salinity to one absolutely dominated by the phylum Euryarchaeota at high salinity. Carbon cycling gene profiles differed significantly among salinity groups: Calvin cycle and rTCA cycle gene abundances were higher in carbon fixation pathways. Nitrogen mineralization and ANRA gene abundances were significantly higher. As salinity increased, transporter and pyrimidine metabolism gene abundances were higher, while the relative contribution of purine metabolism genes decreased, reflecting adaptive adjustments by microorganisms to phosphorus acquisition and nucleic acid biosynthesis under high-salinity stress. The co-occurrence network analysis revealed dense positive associations among functional genes involved in the carbon, nitrogen, and phosphorus cycles. The network analysis identified mer, GLU, and ppk1 as the most highly connected hub genes. Mantel tests indicated that salinity and pH are the primary environmental factors associated with changes in functional gene profiles. This study systematically reveals, at the metagenomic level, how salinity is associated with functional genes involved in carbon, nitrogen, and phosphorus cycles and with microbial community structure. It elucidates the coupling relationships among different elemental cycles, providing a theoretical basis for understanding how biogeochemical cycles in salt lake ecosystems respond to salinization, as well as a scientific reference for predicting the evolutionary trends of inland water ecosystem functions under salinization conditions.

## Data Availability

The original contributions presented in this study are included in the article. Further inquiries can be directed to the corresponding authors.

## Appendix A

**Table A1:** Environmental parameters of low salinity water, medium salinity water, and high-salinity water samples. Values are presented as mean ± standard deviation.

Environmental Parameters	Low Salinity	Medium Salinity	High Salinity
pH	8.88 ± 0.13	8.41 ± 0.33	8.13 ± 0.26
Salinity	4.45 ± 3.63	15.41 ± 2.82	25.24 ± 4.61
TP (μmol/L)	3.30 ± 0.16	5.84 ± 2.06	5.37 ± 1.63
DOC (mg/L)	2.98 ± 1.17	5.66 ± 1.93	4.01 ± 1.57
TN (mg/L)	1.40 ± 0.29	3.68 ± 2.02	2.91 ± 2.12

**Table A2:** Information on microbial functional genes involved in the C, N, and P cycling processes identified in this study.

Gene Category	Subcategory	KEGG Orthology Number	Encoded Protein (EC)/KO Description	Gene Name
C cycle	Starch	K00705	EC:2.4.1.25	*malQ*
		K01176	EC:3.2.1.1	*amyA*
		K01187	EC:3.2.1.20	*malZ*
	Hemicellulose	K01191	EC:3.2.1.24	*MAN2C1*
		K01195	EC:3.2.1.31	*uidA*
		K01198	EC:3.2.1.37	*xynB*
		K01209	EC:3.2.1.55	*abfA*
		K01218	EC:3.2.1.78	*gmuG*
		K01805	EC:5.3.1.5	*xylA*
		K10543	D-xylose transporter	*xylF*
		K10544	D-xylose transport system permease protein	*xylH*
	Pectin	K01728	EC:4.2.2.2	*pel*
	Cellulose	K01222	EC:3.2.1.86	*celF*
		K05349	EC:3.2.1.21	*bglX*
		K05350	EC:3.2.1.21	*bglB*
	Chitin	K01183	EC:3.2.1.14	*chi*
	Lignin	K00432	EC:1.11.1.9	*gpx*
		K03386	EC:1.11.1.24	*Peroxiredoxin*
		K03862	EC:1.14.13.82	*vanA*
	Multiple systems	K01962	EC:2.1.3.15;6.4.1.2	*accA*
		K14534	EC:4.2.1.120 5.3.3.3	*abfD*
		K11263	EC:6.4.1.2 6.4.1.3 6.4.1.- 6.3.4.14	*pccA*
	rTCA cycle	K00031	EC:1.1.1.42	*icd*
		K00170	EC:1.2.7.1	*porB*
		K00169	EC:1.2.7.1	*porA*
		K00024	EC:1.1.1.37	*mdh*
		K01681	EC:4.2.1.3	*ACO*
		K00174	EC:1.2.7.11	*korA*
		K00175	EC:1.2.7.11	*korB*
	Reductive acetyl—CoA pathway	K01938	EC:6.3.4.3	*fhs*
		K07321	carbon monoxide dehydrogenase accessory	*cooC*
	Calvin cycle	K01601	EC:4.1.1.39	*rbcL*
		K01602	EC:4.1.1.39	*rbcS*
		K00615	EC:2.2.1.1	*tktA*
		K00134	EC:1.2.1.12	*GAPDH*
		K00855	EC:2.7.1.19	*prk*
		K03841	EC:3.1.3.11	*fbp*
	CO oxidation	K03518	EC:1.2.5.3	*coxS*
		K03519	EC:1.2.5.3	*coxM*
		K03520	EC:1.2.5.3	*coxL*
	Methane oxidation	K10945	methane/ammonia monooxygenase subunit B	*pmoB-amoB*
	Methanogen	K00577	EC:7.2.1.4	*mtrA*
		K00201	EC:1.2.7.12	*fwdB*
		K03388	EC:1.8.7.3 1.8.98.4 1.8.98.5 1.8.98.6	*hdrA2*
		K03389	EC:1.8.7.3 1.8.98.4 1.8.98.5 1.8.98.6	*hdrB2*
		K03390	EC:1.8.7.3 1.8.98.4 1.8.98.5 1.8.98.6	*hdrC2*
		K00399	EC:2.8.4.1	*mcrA*
		K00320	EC:1.5.98.2	*mer*
		K01499	EC:3.5.4.27	*mch*
		K14083	EC:2.1.1.250	*mttB*
N cycle	Nitrogen fixation	K02586	EC:1.18.6.1	*nifD*
		K02588	nitrogenase iron	*nifH*
		K02591	EC:1.18.6.1	*nifK*
	Nitrification	K10535	EC:1.7.2.6	*hao*
	Denitrification	K00370	EC:1.7.5.1;1.7.99.-	*narG*
		K00371	EC:1.7.5.1;1.7.99.-	*narH*
		K00374	EC:1.7.5.1;1.7.99.-	*narI*
		K00368	EC:1.7.2.1	*nirK*
		K15864	EC:1.7.2.1;1.7.99.1	*nirS*
		K04561	EC:1.7.2.5	*norB*
		K02305	nitric oxide reductase subunit C	*norC*
		K00376	EC:1.7.2.4	*nosZ*
	Assimilatory nitrate reduction (ANRA)	K10534	EC:1.7.1.1 1.7.1.2 1.7.1.3	*NR*
		K00360	EC:1.7.99.-	*nasB*
		K00366	EC:1.7.7.1	*nirA*
		K00367	EC:1.7.7.2	*narB*
	Dissimilatory nitrate reduction (DNRA)	K00362	EC:1.7.1.15	*nirB*
		K00363	EC:1.7.1.15	*nirD*
		K03385	EC:1.7.2.2	*nrfA*
		K15876	nitrite reductase complex	*nrfH*
		K02567	EC:1.9.6.1	*napA*
		K02568	Periplasmic nitrate reductase, electron transfer subunit	*napB*
	Nitrogen mineralization	K00262	EC:1.4.1.4	*gdhA*
		K15371	EC:1.4.1.2	*GDH2*
		K00260	EC:1.4.1.2	*gudB*
		K01725	EC:4.2.1.104	*cynS*
		K00926	EC:2.7.2.2	*arcC*
	Nitrogen assimilation	K00265	EC:1.4.1.13	*gltB*
		K00266	EC:1.4.1.13	*gltD*
		K01915	EC:6.3.1.2	*glnA*
		K00284	EC:1.4.7.1	*GLU*
		K00264	EC:1.4.1.14	*GLT1*
P cycling	Organic phosphoester hydrolysis	K01113	EC:3.1.3.1	*phoD*
		K01077	EC:3.1.3.1	*phoA*
	Inorganic P solubilization	K01507	EC:3.6.1.1	*ppa*
		K00937	EC:2.7.4.1	*ppk1*
	Pentose phosphate pathway	K01807	EC:5.3.1.6	*rpiA*
		K01839	EC:5.4.2.7	*deoB*
		K00033	EC:1.1.1.44 1.1.1.343	*gnd*
		K00948	EC:2.7.6.1	*prsA*
	Phosphonate and phosphinate metabolism	K05781	putative phosphonate transport system ATP-binding	*phnK*
		K19670	EC:3.11.1.2	*phnA*
		K05774	EC:2.7.4.23	*phnN*
		K05780	EC:2.7.8.37	*phnL*
		K06164	EC:2.7.8.37	*phnI*
		K05306	EC:3.11.1.1	*phnX*
	Phosphotransferase system	K02784	phosphocarrier	*ptsH*
	Purine metabolism	K00939	EC:2.7.4.3	*adk, AK*
		K01939	EC:6.3.4.4	*purA*
		K01951	EC:6.3.5.2	*guaA*
		K00602	EC:2.1.2.3 3.5.4.10	*purH*
		K01589	EC:6.3.4.18	*purK*
		K01933	EC:6.3.3.1	*purM*
		K23269	EC:6.3.5.3	*purL*
		K00940	EC:2.7.4.6	*ndk*
		K11751	EC:3.1.3.5 3.6.1.45	*ushA*
	Pyrimidine metabolism	K00943	EC:2.7.4.9	*tmk*
		K01520	EC:3.6.1.23	*dut*
		K21636	EC:1.1.98.6	*nrdD*
		K00945	EC:2.7.4.25	*cmk*
		K01591	EC:4.1.1.23	*pyrF*
	Pyruvate metabolism	K01596	EC:4.1.1.32	*pckA*
		K01007	EC:2.7.9.2	*ppsA*
		K01006	EC:2.7.9.1	*ppdK*
	Transporters	K05814	sn-glycerol 3-phosphate transport system permease	*ugpA*
		K05813	sn-glycerol 3-phosphate transport system substrate-binding	*ugpB*
		K05816	EC:7.6.2.10	*ugpC*
		K05815	sn-glycerol 3-phosphate transport system permease	*ugpE*
		K02041	EC:7.3.2.2	*phnC*
		K02044	phosphonate transport system substrate-binding	*phnD*
		K02042	phosphonate transport system permease	*phnE*
		K02036	EC:7.3.2.1	*pstB*
		K02040	phosphate transport system substrate-binding	*pstS*
		K02037	phosphate transport system permease	*pstC*
	P-starvation response regulation	K07657	phosphate regulon response	*phoB*
		K02039	phosphate transport system	*phoU*
		K07636	EC:2.7.13.3	*phoR*
